# Parturient on Magnesium Infusion and Its Effectiveness as an Adjuvant Analgesic after Cesarean Delivery: A Retrospective Analysis

**DOI:** 10.1155/2018/3978760

**Published:** 2018-11-15

**Authors:** Tanmay H. Shah, Abby R. Rubenstein, Edward S. Kosik, Stephen W. Heimbach, Abhinava S. Madamangalam

**Affiliations:** ^1^Assistant Professor, MD, MBBS, University of Oklahoma Health Sciences Center, Oklahoma City, OK, USA; ^2^Resident in Obstetrics & Gynecology Department, MD, University of Oklahoma Health Sciences Center, Oklahoma City, OK, USA; ^3^Associate Professor, DO, University of Oklahoma Health Sciences Center, Oklahoma City, OK, USA; ^4^Staff Anesthesiologists, MD, Affiliated Anesthesiologists, Oklahoma City, OK, USA; ^5^Faculty Anesthesiologist, MD, Geisinger Medical Center, Danville, PA, USA

## Abstract

**Background:**

Perioperative use of intravenous magnesium as part of multimodal analgesia has been increasing in recent years in an effort to decrease the use of opioids. The aim of this study was to evaluate the effectiveness of magnesium sulfate infusion in lowering analgesic requirement and decreasing the intensity of pain score after cesarean delivery.

**Methods:**

Sixty-four patients who underwent cesarean delivery under spinal anesthesia were included in this medical record review: 32 patients received magnesium infusion after cesarean delivery for treatment of mild preeclampsia (Mg group); 32 patients received routine post-cesarean delivery care (control group). Primary outcome was total analgesic consumption and secondary was visual analogue scores (VAS) of pain in each group during the first 24 hours following delivery. These measures were compared using Student's t-tests and Mann-Whitney U-tests.

**Results:**

Our study found that patients in the Mg group had significantly less requirement for analgesia than the control group. In the 24 h after cesarean delivery, the Mg group received significantly less intravenous ketorolac (the standard initial rescue analgesic agent) when compared to the control group (79 ± 23 mg vs. 90 ± 0 mg;* P* = 0.008). The Mg group also received significantly less intravenous morphine equivalents than the control group (median 5.0 (IRQ: 0.0 – 10.0) vs. 9.3 (IRQ: 6.0 – 21.1);* P* = 0.001) during the first 24 h after cesarean delivery. The Mg group also had significantly lower VAS pain scores than the control group (median 1.75 (IRQ: 0.4 – 2.6) vs. median 3.2 (IRQ: 2.3 – 4.5);* P *< 0.001).

**Conclusions:**

Our results suggest that magnesium sulfate infusion decreases total analgesic requirements and lowers VAS pain scores during the first 24 h after cesarean delivery.

## 1. Introduction

Cesarean delivery is one of the most common surgical procedures performed in the United States. According to the Centers for Disease Control and Prevention, 32.2% of births were by cesarean delivery in 2014 [1] compared to 4.5% in 1965 [2]. An increased cesarean delivery rate has led to more women being treated for acute post-cesarean pain or scar hyperalgesia after previous cesarean delivery. Inadequately treated acute pain is known to contribute to chronic postoperative pain and other adverse sequelae. Although multimodal analgesic strategies, including wound infiltration with a local anesthetic agent, intravenous (IV) ketamine, and transversus abdominis plane blocks, are now being used to decrease acute pain after cesarean delivery, many patients still develop acute and chronic postoperative pain. Currently, the incidence of chronic pain after cesarean delivery ranges from 1% to 18% [3]. Recent reports have also shown development of posttraumatic stress disorder in women who experience chronic pain, which can affect maternal quality of life postpartum [4].

Intravenous opioids are routinely used as rescue analgesics to attenuate acute postoperative pain. One nonopioid medication now attracting attention as a potential adjuvant analgesic for treatment of acute postoperative pain is magnesium sulfate. However, there is limited information in the current literature concerning the use and effectiveness of magnesium as an adjuvant analgesic in the peripartum period.

While a number of studies have focused on the effectiveness of magnesium in decreasing postoperative pain and analgesic requirements, few have evaluated the analgesic effect of magnesium sulfate during post-cesarean delivery with inconclusive results [5]. We hypothesized that magnesium sulfate could be a useful adjuvant analgesic in the postoperative period after cesarean delivery.

Obstetricians often use magnesium sulfate infusion during labour in patients with preeclampsia to prevent its progression to eclampsia. Furthermore, these patients routinely receive magnesium sulfate infusion for 24 h after delivery. In this study, we assessed the ability of this existing therapeutic obstetric measure to decrease maternal analgesic requirements and to reduce the intensity of acute pain in the first 24 h after cesarean delivery. Our hypothesis was that there would be a reduction in total analgesic requirement and in VAS pain scores during the first 24 hours after cesarean delivery if parturient received magnesium infusion during that period.

## 2. Materials and Methods

This study was approved by University of Oklahoma Health Sciences Center Institutional Review Board (IRB). The need for written informed consent was waived by IRB due to retrospective nature of the research. The manuscript adheres to the applicable Equator guidelines.

We reviewed the medical records of patients who had undergone cesarean delivery with spinal anesthesia at the Women's and Newborn Center of University of Oklahoma Health Sciences Center between January 1, 2014, and September 30, 2014. All patients that had singleton pregnancies who were scheduled for elective cesarean delivery and received a standard dose of spinal anesthesia (12 mg of hyperbaric bupivacaine along with 20 mcg of fentanyl and 200 mcg of morphine intrathecally) were considered. Patients were excluded if they obtained inadequate anesthesia following spinal block for cesarean delivery and required conversion to general anesthesia or supplementation with intravenous analgesics. Patients who received transversus abdominis plane blocks for additional pain relief were also excluded. The magnesium group (Mg group) included patients who received magnesium sulfate infusion for at least 20 h after cesarean delivery; all of them were treated for preeclampsia with no features of severe preeclampsia.

Bolus administration of magnesium sulfate was not given; all patients in the Mg group received 2 g/hr of magnesium infusion after cesarean delivery. None of these patients had severe preeclampsia or any other comorbidity. In the control group, there was no administration of magnesium infusion and all the patients were healthy without any comorbidity. Patients who met the eligibility criteria were selected from the available electronic medical records.

Approximately one patient was randomly selected for each group from each week of the 9-month study period and from alternating operating room logs kept by the obstetric anesthesia division. The sample size of 32 patients in each group was estimated to be able to show a 2-point (20%) difference in mean VAS score between the two groups. Sixty-four patients were selected, comprising 32 in the Mg group and 32 in the control group. None of these patients received magnesium before or during cesarean delivery.

Data extracted from each patient's medical records were age, height, weight, gestational age, race, gravida, parity, timing of intrathecal opioid administration, time of arrival in the postanesthesia care unit, and whether the procedure was a primary or repeat cesarean delivery. Time of arrival in the postanesthesia care unit was designated as t0 and all medications given for pain relief within 24 h following t0 were recorded. Analgesics administered by the obstetric anesthesia division in the first 24 h after delivery were mostly IV ketorolac (scheduled) and IV fentanyl (as needed).

Visual analog scale (VAS) for pain was recorded whenever available in the medical record. The VAS score ranges from 0 (no pain) to 10 (worst imaginable pain). VAS score was not consistently recorded, as nurses are only required to record the VAS score when administering any type of analgesia. For analysis, all analgesics and VAS score data were divided into four 6 h periods: 0–6 h, 6–12 h, 12–18 h, and 18–24 h after t0. Analgesics received by the patients were considered both individually and as groups, e.g., opioids or nonsteroidal anti-inflammatory drugs. In addition, all opioids were converted into IV morphine equivalents to determine the total analgesic requirements of the patients in the first 24 h after cesarean delivery.

## 3. Statistical Analysis

The primary outcome of the study was the total analgesic requirement in the first 24 h after cesarean delivery and the secondary outcome was the VAS score for pain during the same period. For the demographics (Table 1) and intravenous ketorolac requirement (Table 2) group statistics were reported as mean ± standard deviation for continuous measures and as percentages for counts. Student's* t*-tests were used to compare means for continuous measures. Pearson chi-squared tests were used to compare distributions of counts. The sampling distribution of measures of morphine requirement (Table 3) and VAS pain score (Table 4) were skewed in some cases so group statistics were reported as median, interquartile range (25^th^ and 75^th^ percentiles) and range (minimum and maximum). Mann-Whitney U-tests were used to compare groups. The correlations in outcome measures between one time-period and the next were very small (absolute correlation, r <0.02), so that adjusting for repeated measures made no difference to the results. A Holm-Bonferroni correction was used to correct for multiple comparisons of groups over several time-periods. The sample size of 32 patients in each group was estimated to be able to show a 2-point (20%) difference in mean VAS score between the two groups with a significance level of 0.05% and 90% power. The primary and repeat cesarean groups were also analyzed separately and compared to determine if differences in proportions of primary versus repeat cesarean delivery might contribute to the differences seen in the primary and secondary outcomes between the study groups. The statistical analysis was performed using SPSS for Windows version 20.0 (2011) software (IBM Corp., Armonk, NY, USA). A* P*-value <0.05 was considered to be statistically significant.

## 4. Results

The demographics and pregnancy-related characteristics of the women in the two study groups are shown in Table 1. There were no significant differences in age, race, height, or weight between the two groups. However, there were significant differences in gestational age and the proportions of primary vs. repeat cesarean deliveries between the study groups (*P *< 0.05). Gestational age was significantly lower in the magnesium group than in the control group (34.6 ± 3.6 weeks vs. 38.9 ± 1.3 weeks;* P *< 0.001). This is likely to be a consequence of all patients in the Mg group having preeclampsia since women with this condition tend to require cesarean delivery at an earlier gestational age than normal.

Forty-one percent of the patients in the Mg group had repeat cesarean delivery compared with 69% in the control group. Repeat cesarean delivery can potentially change the severity of postoperative pain and analgesic requirements. To avoid potential confounding, we separated patients undergoing a primary procedure and those undergoing a repeat procedure and repeated the analyses on each group separately. Similar results were obtained in each group with regard to the decrease in total analgesic consumption and VAS scores in the Mg group. Therefore, whether the procedure was primary or repeat was not a confounding factor.

## 5. Primary Outcome

Total analgesic requirements during the first 24 h after cesarean delivery were compared between the two study groups. Nonsteroidal anti-inflammatory drugs were considered separately from opioid analgesics.

### 5.1. IV Ketorolac

IV ketorolac is administered every 6–8 h in the first 24 h after cesarean delivery at our institution depending on the patient's need. The Mg group required, on average, just over 10 mg less IV ketorolac than the control group during the first 24 h postoperatively; the difference was statistically significant (*P *= 0.008; Table 2). In general, fewer patients in the Mg group required IV ketorolac in each of the 6 h periods than in the control group. The largest (and significant) differences were found in the 6–12 h (*P *= 0.001) and 12–18 h (*P *= 0.013) times. Similar results were found when patients with primary and repeat cesarean delivery were analyzed separately.

### 5.2. IV Opioids

**Figure 1:**
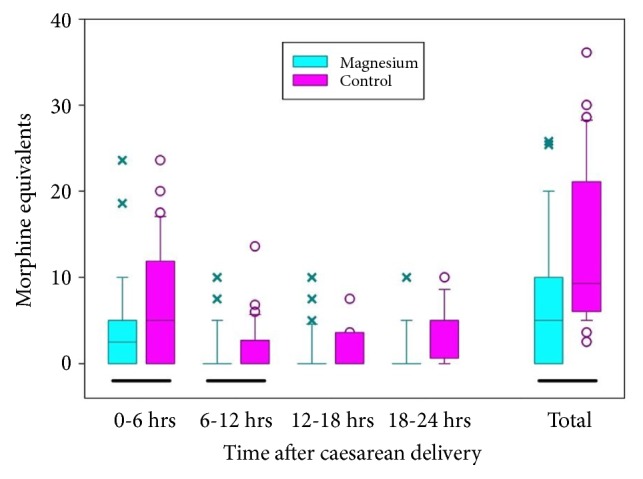
Intravenous morphine equivalent requirement at different time intervals. Statistically significant at 0–6 h and 18–24 h, as well as for total requirement. Box shows the interquartile range, horizontal line within box shows median, whiskers show 10/90th percentiles, x and o show points outside of the 10/90th percentiles. Solid bar beneath pairs of groups indicates a significant difference between the groups (p < 0.05).

IV fentanyl was the most common opioid medication used as rescue analgesics when pain was not satisfactorily controlled by IV ketorolac. Patients in the Mg group required about half the total opioid dose (in IV morphine equivalents; Table 3) in the 24 h after cesarean delivery when compared with that required in the control group (*P *= 0.001), and they required a consistently lower opioid dose across all four 6 h periods, particularly at 18–24 h (*P *< 0.001; Figure 1).

## 6. Secondary Outcome

**Figure 2:**
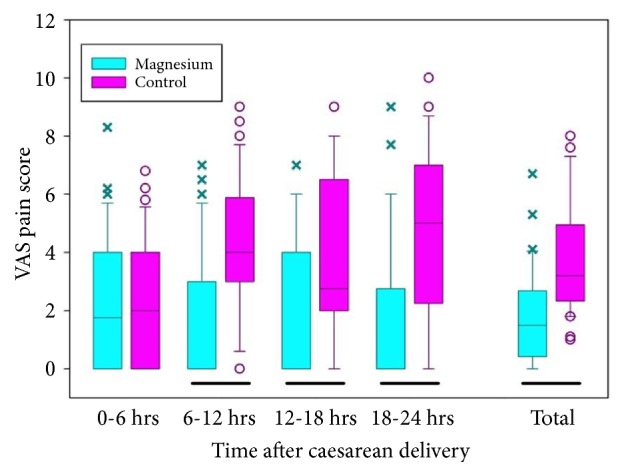
Average pain scores in the magnesium and control groups. Box shows the interquartile range, horizontal line within box shows median, whiskers show 10/90th percentiles, x and o show points outside of the 10/90th percentiles. Solid bar beneath pairs of groups indicates a significant difference between the groups (p < 0.05).

**Figure 3:**
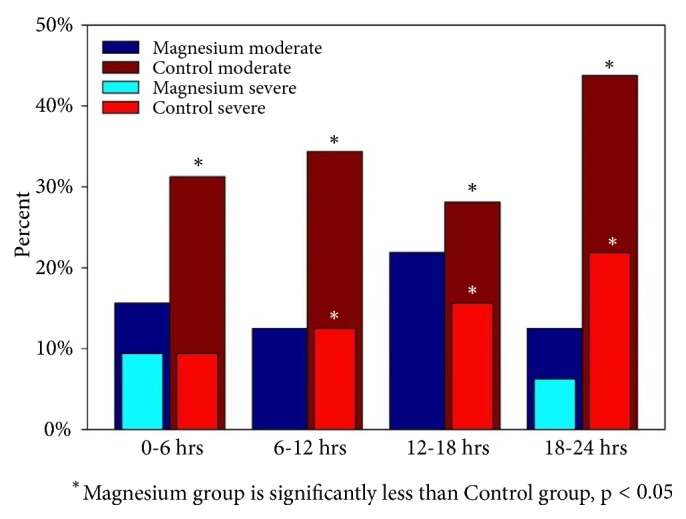
Proportions of patients registering moderate to severe pain in the magnesium and control groups.

The average VAS score in the 24 h after delivery was significantly lower (nearly half) in the Mg group when compared with the control group (*P *< 0.001; Table 4). The patients in the Mg group experienced significantly less pain following delivery than the control group, especially from 6 h onwards (Figure 2). The results were similar when the VAS scores were analyzed according to whether cesarean delivery was a primary or repeat procedure. To confirm that the high average VAS scores in the control group were not an artifact of a small minority of patients experiencing very high levels of pain, the VAS scores were categorized as low (0–3), moderate (4–6), or severe (7–10).  Figure 3 shows that patients in the control group experienced more moderate or severe pain whereas no patient in the Mg group experienced severe pain in the 6–12 h or 12–18 h postoperative periods.

## 7. Discussion

This study shows that magnesium sulfate infusion postoperatively decreases the requirement for total analgesics and reduces pain scores in the first 24 h following cesarean delivery.

Magnesium sulfate, a nonopioid analgesic, is being used as an adjuvant perioperative analgesic at various institutions in their Perioperative Surgical Home (PSH) initiative. It has shown benefit after major orthopedic surgery [6], abdominal surgery [7–9], and lower limb orthopedic surgery [10]. While magnesium is known to be a noncompetitive N-methyl-D-aspartate receptor antagonist that prevents the calcium influx leading to a change in the intracellular calcium concentration [11], the basic mechanism for the analgesic effect of magnesium is still unclear. It is presumed that magnesium can prevent central sensitization secondary to the generation of noxious peripheral stimuli and blunt the hypersensitivity response to nociception. This is believed to be the mechanism leading to a change in the perception of pain.

Intrathecal opioid administration during cesarean delivery is considered the standard of care and usually provides analgesia for the initial 24 h after delivery; however, many patients still experience significant breakthrough pain during this time. This can potentially affect maternal and infant bonding, breast-feeding, and maternal recovery. Our study suggests that intravenous magnesium sulfate can be a useful adjuvant analgesic in the postoperative period following cesarean delivery.

Albrecht et al. [12] and De Oliveira et al. [13] conducted meta-analyses to determine the effect of magnesium sulfate on postoperative pain and found that magnesium sulfate given as either a bolus or as an infusion reduced postoperative pain and total opioid consumption in the first 24 h postoperatively without any adverse effects in a variety of types of surgery. For cesarean delivery, previous studies have shown conflicting results regarding the ability of perioperative intravenous magnesium to decrease postoperative pain [5, 14, 15]. Rezae et al. [14] evaluated the analgesic effect of preemptive magnesium sulfate infusion on postoperative pain after elective cesarean delivery and showed decreases in cumulative analgesic consumption, postoperative pain scores up to 24 h after delivery, and number of shivering incidents. Mireskandari et al. [15] similarly reported a reduced total analgesic requirement and significantly decreased pain scores when magnesium sulfate was given as an intravenous bolus preoperatively. Our study showed similar benefits of reduced analgesic consumption and reduced pain scores, but for intravenous magnesium given postoperatively as a continuous infusion for 24 h rather than as a preemptive bolus. However, Paech et al. [5] did not find a reduction in the severity of short-term or long-term (6 weeks) pain after cesarean delivery, or in the total analgesic consumption after cesarean delivery, when magnesium sulfate was given as an intravenous infusion postoperatively. Epidural PCA (patient controlled analgesia) was used in Paech's study [5] and it is possible that superior analgesic effect provided by epidural PCA may have masked the analgesic adjuvant effect of magnesium. Lee et al. [16] recommended that magnesium sulfate can be used as an adjuvant during general anesthesia for cesarean delivery to decrease the incidence of perioperative awareness and hemodynamic instability arising from inadequate anesthesia, analgesia, or both. Apan et al. [17] also evaluated the effectiveness of a 24 h intravenous magnesium sulfate infusion in reducing the consumption of analgesics after spinal anesthesia in nonobstetric patients and found that the time to first need for analgesia was increased and total consumption of analgesics was reduced in their magnesium group. Ryu et al. [18] have also shown an improvement in the postoperative analgesia in a group who received magnesium infusion during the gynecological surgery in addition to reduced requirement of rocuronium.

In our study, postoperative infusion of magnesium sulfate was associated with a significantly lower requirement for morphine and nonsteroidal anti-inflammatory drugs during the first 24 h after cesarean delivery. There also was a significant decrease in VAS pain score when compared with the control group, as well as significantly fewer reports of moderate or severe pain in the magnesium group. None of the parturients had side effects related to magnesium infusion such as nausea, vomiting, flushing, decreased deep tendon reflexes, altered mental status, or respiratory depression.

From our study, magnesium sulfate given as an infusion after cesarean delivery appears to reduce the acute postoperative pain and may possibly decrease the incidence of chronic postoperative pain. This suggests that a clinical trial, avoiding the confounding with preeclampsia, is an appropriate next step for further research.

There are some limitations in this study. First, the effect of adjuvant magnesium was confounded with the presence of preeclampsia. As this was not a clinical trial but drew on existing medical records, this was an unavoidable consequence of the treatment protocols in use. This was a retrospective chart review in which magnesium group comprised patients with preeclampsia as magnesium is routinely given in parturient with preeclampsia. Second, and a consequence of the first, there was a significant difference in gestational age at birth between the magnesium and control groups that could not be addressed because this factor was almost completely confounded by simply being in the magnesium or control group. Patients with preeclampsia tend to deliver early. Third, the patient pool was limited to preexisting patients from our hospital and the data collection was limited to what was recorded in the patients' medical records. There was no opportunity to collect additional data, such as VAS scores for each patient at every time interval, since typically no VAS score was recorded if no analgesia was required. Based on our findings we have planned for prospective study that will eliminate these limitations and confounding factor, preeclampsia, which was present in our retrospective analysis, to evaluate the efficacy of magnesium sulfate infusion as an adjuvant analgesic after cesarean delivery.

In conclusion, this study suggests the effectiveness of magnesium sulfate infusion in reducing the total analgesic consumption and decreasing VAS pain scores after cesarean delivery. We suggest that magnesium should be included as a multimodal analgesia in the enhanced recovery pathway for cesarean delivery. However, caution is required when administering an infusion of magnesium sulfate in patients with renal impairment or cardiac conduction abnormalities to avoid adverse events. Future studies should focus on using intravenous magnesium at an intermittent dosing interval rather than as a continuous infusion to investigate its efficacy as an adjuvant analgesic.

## Figures and Tables

**Table 1:** Patient demographics and pregnancy characteristics in the magnesium and control groups. Data presented as the mean ± standard deviation and as percentage for counts.

	Magnesium group	Control group	*P-*value
Age (y)	28.5 ± 5.2	27.3 ± 5.4	0.36
Race (White/Other)	12/20 (38% White)	14/18 (44% White)	0.61
(W/B/A/H/Other)	12/9/3/7/1	14/6/4/8/0	0.81
Height (meters)	1.61 ± 0.07	1.63 ± 0.07	0.26
Weight (kg)	96 ± 21	86 ± 15	0.056
Gestational age (weeks)	34.6 ± 3.6	38.9 ± 1.3	<0.001
Primary/repeat	19/13 (59% primary)	10/22 (31% primary)	0.026

W/B/A/H = White/Black/Asian/Hispanic.

**Table 2:** Intravenous ketorolac requirement in first 24 hours following caesarean delivery in the magnesium and control groups.

	Magnesium group	Control group	*P-*value
Total IV ketorolac (mg)	79 ± 23	90 ± 0	0.008

Use of ketorolac within time period (% yes)			

IV ketorolac 0–6 h	38%	44%	0.39
IV ketorolac 6–12 h	38%	91%	0.001
IV ketorolac 12–18 h	34%	75%	0.013
IV ketorolac 18–24 h	16%	16%	0.66

Data presented as the mean ± standard deviation for continuous measures and as percentage for counts.

**Table 3:** Intravenous morphine requirement in first 24 hours following caesarean delivery^∗^.

	Magnesium group	Control group	*P-*value
Total IV morphine (mg)	5.0 (0.0 – 10.0) [0.0 – 25.8]	9.3 (6.0 – 21.1) [2.5 – 36.1]	0.001

IV morphine 0–6 h (mg)	2.5 (0.0 – 5.0) [0.0 – 23.6]	5.0 (0.0 – 10.0) [0.0 – 23.6]	0.019

IV morphine 6–12 h (mg)	0.0 (0.0 – 0.0) [0.0 – 10.0]	0.0 (0.0 – 0.0) [0.0 – 13.6]	0.57

IV morphine 12–18 h (mg)	0.0 (0.0 – 0.0) [0.0 – 10.0]	0.0 (0.0 – 3.6) [0.0 – 7.5]	0.19

IV morphine 18–24 h (mg)	0.0 (0.0 – 0.0) [0.0 – 10.0]	5.0 (0.0 – 5.0) [0.0 – 10.0]	<0.001

^*∗*^Converted to morphine equivalent dosages for various opioids.

Values expressed as median (InterQuartile Range) [Range].

**Table 4:** Average VAS pain scores in first 24 hours after caesarean delivery.

	Magnesium group	Control group	*P-*value
VAS overall	1.75 (0.4 – 2.6) [0.0 – 6.7]	3.2 (2.3 – 4.5) [1.0 – 8.0]	<0.001

VAS 0–6 h	0.0 (0.0 – 4.0) [0.0 – 8.3]	2.0 (0.0 – 4.0) [0.0 – 6.8]	0.67

VAS 6–12 h	0.0 (0.0 – 3.0) [0.0 – 7.0]	4.0 (3.0 – 5.5) [0.0 – 9.0]	<0.001

VAS 12–18 h	0.0 (0.0 – 4.0) [0.0 – 7.0]	2.75 (2.0 – 6.5) [0.0 – 9.0]	0.003

VAS 18–24 h	0.0 (0.0 – 2.0) [0.0 – 9.0]	5.0 (2.0 – 7.0) [0.0 – 10.0]	<0.001

Abbreviation: VAS, visual analog scale

Values expressed as median (InterQuartile Range) [Range].

## Data Availability

The data used to support the findings of this study are restricted by the Institutional Review Board at the University of Oklahoma Health Sciences Center in order to protect patient privacy. Data are available from the corresponding author, Tanmay Shah, for researchers who meet the criteria for access to confidential data. The corresponding author will initiate the data sharing process with ORA (Office of Research Administration) at the University of Oklahoma Health Sciences Center after receiving the request.
